# Medication-Documented Systemic Anticancer Treatment in Colorectal Cancer: A Single-Centre Routine-Data Study

**DOI:** 10.3390/jcm15218486

**Published:** 2026-10-31

**Authors:** Alexandru-Marian Vieru, Radu Meșină, Virginia-Maria Rădulescu, Emil-Tiberius Trașcă, Sergiu-Marian Cazacu, Maria-Lorena Mustață, Daniela-Maria Micu, Tudor-Adrian Bălșeanu, Petrică Popa, Tudorel Ciurea

**Affiliations:** 1 Doctoral School, University of Medicine and Pharmacy of Craiova, 200349 Craiova, Romania; alexandruvieru1993@gmail.com (A.-M.V.); umlorena@yahoo.com (M.-L.M.); 2 Faculty of Medicine, University of Medicine and Pharmacy of Craiova, 200349 Craiova, Romania; virginia.radulescu@umfcv.ro (V.-M.R.); etrasca@yahoo.com (E.-T.T.); sergiu.cazacu@umfcv.ro (S.-M.C.); daniela.micu@umfcv.ro (D.-M.M.); adibalseanu@yahoo.com (T.-A.B.); popa.petrica@umfcv.ro (P.P.); tudorel.ciurea@umfcv.ro (T.C.); 3 Research Centre for Gastroenterology and Hepatology, 200349 Craiova, Romania

**Keywords:** colorectal cancer, systemic anticancer therapy, real-world data, treatment patterns, treatment exposure, targeted therapy, administrative data, concordance

## Abstract

**Background/Objectives:** Real-world colorectal cancer (CRC) treatment is documented across multiple routine hospital data domains, which complicates interpretation of treatment-pattern studies. We characterised medication-documented systemic anticancer treatment patterns in CRC and evaluated the consistency of the routine data sources used to ascertain them. **Methods**: This retrospective observational study included 1310 encounters from 627 patients with C18—C20 diagnosis codes from 1 March to 30 April 2026. Semicolon-delimited medication entries were normalised to named agents. Patient-level medication-derived treatment patterns were characterised among 54 patients with at least one named antineoplastic documented during the fixed study window, without assuming representativeness of the full CRC-coded cohort; oral prescriptions were analysed separately as complementary evidence. **Results**: Twenty patients (37.0%) had cytotoxic component(s) only, 20 (37.0%) targeted/immune-checkpoint-inhibitor (ICI) plus cytotoxic components, 12 (22.2%) targeted/ICI components without cytotoxic co-documentation, and 2 (3.7%) other antineoplastic agents only. At the first treatment-documented encounter, 24/54 (44.4%) had at least two named antineoplastic agents co-documented. Thirty-three patients (61.1%) had medication-positive treatment documentation in at least two encounters, and 30/33 (90.9%) had the same documented class set across these encounters. Procedure- and medication-based indicators agreed in 890/891 interpretable encounters (99.89%; Cohen’s kappa 0.9953, 95% CI 0.9839–1.0000). **Conclusions**: Routine hospital medication data showed heterogeneous medication patterns among CRC-coded patients, frequently with multi-component and repeated documentation. Procedure and medication indicators share a hospital workflow; their near-complete concordance describes internal consistency and cannot exclude joint under-capture or establish clinical accuracy or treatment appropriateness.

## 1. Introduction

Colorectal cancer (CRC) remains one of the most common malignancies worldwide and is a major cause of cancer mortality. Global estimates for 2022 attributed approximately 9.6% of incident cancers and 9.3% of cancer deaths to colorectal malignancies [1]. Systemic treatment is central to CRC care across multiple clinical settings, with contemporary treatment strategies incorporating fluoropyrimidines, oxaliplatin, irinotecan, anti-vascular endothelial growth factor (VEGF) therapy, anti-epidermal growth factor receptor (EGFR) therapy, immune checkpoint inhibition, and oral agents used in selected clinical contexts [2,3,4].

Randomised trials establish treatment efficacy, but routine clinical practice includes broader and more heterogeneous populations, treatment sequences, and documentation processes. Real-world data (RWD) therefore provide an important complementary perspective on treatment utilisation and clinical practice in CRC [5,6]. Large contemporary cohorts such as PROMETCO illustrate the value of real-world treatment-pattern research by characterising exposure to fluoropyrimidines, oxaliplatin, irinotecan, anti-VEGF therapy, and anti-EGFR therapy across the metastatic CRC continuum of care [7]. However, treatment-pattern estimates from different RWD sources are not directly comparable unless exposure definitions, observation windows, and source provenance are described explicitly.

Treatment ascertainment is itself a methodological challenge in oncology RWD. A recent CRC-specific scoping review found that definitions of oncological drug exposure were frequently incompletely reported, limiting reproducibility and interpretation [8]. Dedicated structured resources such as the English Systemic Anti-Cancer Therapy (SACT) dataset capture patient-, regimen-, cycle-, and drug-level treatment information [9]. In less structured routine data environments, systemic treatment may instead be represented by procedure codes, medication-administration fields, prescriptions, pharmacy data, free text, or combinations of these sources. Studies using routinely collected data have shown that different sources may capture overlapping but non-identical treatment information, particularly for oral therapy [10,11,12].

We therefore examined CRC treatment patterns as the main descriptive focus and treatment ascertainment as a supporting methodological component. The main objective was to characterise patient-level documented systemic anticancer treatment patterns during a fixed two-month observation window among patients with coded CRC receiving routine hospital care. Secondary descriptive objectives were to characterise medication-derived therapeutic components, same-encounter co-documentation, treatment-pattern complexity, repeated treatment documentation, and oral antineoplastic prescriptions as a separate complementary source. Variation by colorectal tumour-site group was explored, and concordance between procedure-based and medication-based treatment indicators was quantified to assess internal consistency of exposure ascertainment within a shared hospital workflow.

## 2. Materials and Methods

### 2.1. Study Design and Data Source

This retrospective observational study used routinely collected hospital data from Spitalul Clinic de Urgență Militar “Dr. Ștefan Odobleja” Craiova, Romania. Information was abstracted manually from patient records into Excel. Eligible contacts arose from oncology, internal medicine, and surgery. The study window was fixed at 1 March–30 April 2026 because access to the study data was restricted to this period; records outside this interval were excluded and were not analysed. Care included treatment, clinical reviews, investigations and monitoring, and could extend to other centres; complete cross-centre treatment histories were unavailable. Within the study window, 1310 encounters carried an admission diagnosis code in the C18–C20 range and were retained. The dataset contained demographic information, admission and discharge information, diagnosis codes, procedure fields, medication entries, and a separate prescription field. It did not contain sufficiently complete tumour stage, histopathology, molecular profile, treatment intent, treatment line, response, recurrence, or survival outcomes for the present cohort; none of these variables were inferred.

The treatment-pattern research question was developed after an initial audit of the available routine data. The study window, medication-classification rules and derived-variable definitions used in the reported analysis are described below and in the Supplementary Methods. Treatment-pattern measures and tumour-site comparisons are descriptive or exploratory; prospective pre-specification is not claimed.

### 2.2. Study Population and Patient-Level Linkage

All encounters in oncology, internal medicine, or surgery dated within the fixed study window and carrying a C18, C19, or C20 admission diagnosis code were included. A diagnosis code was treated as evidence of a routinely coded CRC diagnosis and not as histopathological confirmation. Repeated encounters were retained. Patient records were linked internally using the national identifier (CNP) as the primary key. After linkage and data cleaning, 627 unique patients were assigned study identifiers and direct identifiers were removed from the analytical files. Eligibility was based on the admission diagnosis field; inclusion using C18–C20 codes in any diagnosis position was not evaluated. Encounters coded primarily with chemotherapy-session or metastatic-site codes may therefore have been missed.

Patient-level tumour-site group was assigned from encounter-level C18–C20 codes using the modal site across observed encounters, with the first chronological site used as a tie-breaker. Eighteen of 627 patients had more than one C18–C20 site group across encounters; these patients remained in the primary analysis, and their exclusion was examined in a sensitivity analysis for site-specific treatment comparisons. Age at the first observed encounter was available for 625 patients; two values could not be determined because the national-identifier century component was not interpretable under the specified derivation rule; no century was assumed. The youngest recorded age, 17 years, was verified as correct by the study team.

### 2.3. Medication and Prescription Exposure Construction

The encounter-level medication field contained semicolon-delimited product entries. Each entry was split into individual tokens, normalised for case, spacing, diacritics, spelling variants, and product naming, and mapped to an active agent where the product label allowed unambiguous identification. Across the study encounters, 5075 medication tokens representing 186 distinct raw token forms were processed. Supportive medicines, intravenous fluids, and devices were not classified as antineoplastic therapy. Calcium folinate/leucovorin was classified as a treatment modulator and was excluded from antineoplastic agent counts. The complete mapping dictionary is provided in Table S2. The rule-based classification was reviewed by an oncologist. Medication entries were recorded in a single hospital field; this recording arrangement does not establish complete capture of every administered component. No independent validation against pharmacy or medication-administration records was performed.

The named antineoplastic set observed in the medication field included fluorouracil, oxaliplatin, irinotecan, bevacizumab, cetuximab, panitumumab, pembrolizumab, carboplatin, etoposide, and gemcitabine. For medication-derived CRC treatment descriptions, the CRC-relevant component set comprised fluorouracil, oxaliplatin, irinotecan, anti-VEGF therapy (bevacizumab), anti-EGFR therapy (cetuximab or panitumumab), and immune checkpoint inhibition (pembrolizumab in the observed data). Carboplatin, etoposide, and gemcitabine were retained as observed antineoplastic exposures but were flagged as outside this CRC-relevant medication-derived set; no treatment indication was inferred for these agents. Membership of this component set denotes potential CRC relevance, not confirmation that the drug was prescribed for CRC in an individual patient.

The separate prescription field was analysed as a complementary source for oral antineoplastic therapy. Capecitabine, trifluridine-tipiracil, and regorafenib were searched separately in this field. A prescription was not treated as proof of drug administration and did not redefine the primary medication-derived treatment pattern or medication-derived therapeutic-class counts. Where medication and prescription evidence were combined in a sensitivity or source-complementarity analysis, this was labelled explicitly. This was a targeted search for these three oral agents, not an exhaustive search of all oral agents relevant to CRC; no inference of absence is made for agents outside this search set.

### 2.4. Derived Systemic Treatment Patterns

The main oncological analysis was conducted at patient level among patients with at least one named antineoplastic medication documented during the study window. Four mutually exclusive medication-derived patterns were defined for the reported descriptive analysis: (1) cytotoxic component(s) only; (2) targeted or immune-checkpoint-inhibitor (ICI) component(s) plus cytotoxic component(s); (3) targeted/ICI component(s) without cytotoxic co-documentation; and (4) other antineoplastic treatment only. Cytotoxic components comprised fluorouracil, oxaliplatin, and irinotecan. Targeted/ICI components comprised bevacizumab, cetuximab, panitumumab, and pembrolizumab. The phrase ‘without cytotoxic co-documentation’ describes the observed medication record only and was not interpreted as confirmed targeted monotherapy. Incomplete recording of a partner drug can move an encounter or patient between these documentation categories; the categories do not establish the complete treatment received.

Additional derived variables included the number of named antineoplastic agents co-documented in the same encounter, the number of distinct medication-derived CRC-relevant therapeutic classes documented per patient during the fixed window, and the number of medication-positive treatment encounters per patient. The earliest medication-positive encounter in the study window was designated the ‘first treatment-documented encounter’; this construct was not interpreted as treatment initiation or first-line therapy. For patients with at least two medication-positive encounters, the documented therapeutic-class set was classified as unchanged if the exact set of medication-derived classes was identical across all such encounters and changed otherwise. Same-encounter component combinations were reported descriptively and were not assigned regimen names such as FOLFOX, FOLFIRI, or CAPOX.

### 2.5. Exploratory Tumour-Site Analysis

Among patients with documented antineoplastic exposure, six medication-derived components were compared between C18 (colon) and C19-C20 (rectosigmoid junction/rectum) patient-level site groups: fluorouracil, oxaliplatin, irinotecan, anti-VEGF, anti-EGFR, and ICI. This analysis was explicitly exploratory. C18 versus C19-C20 was not interpreted as biological right- versus left-sided CRC. Because molecular eligibility, metastatic status, treatment intent, and treatment line were unavailable, site-group differences were not interpreted as evidence of treatment appropriateness or guideline adherence.

### 2.6. Supporting Treatment-Ascertainment Analysis

A supporting agreement analysis compared a selected group of chemotherapy-administration codes within the Romanian RO DRG R001 family (R00101, R00102, R00103, R00107 and R00108), used as a procedure-based indicator of treatment documentation, with the presence of a named antineoplastic agent in the encounter medication field. Code descriptions and analytical roles are provided in Table S1. Both indicators originated from the same encounter record and were not independent reference sources. The primary agreement analysis was restricted to encounters with a nonblank medication field. Blank medication fields were treated as unavailable and not as evidence of no treatment. A separate operational full-cohort sensitivity analysis is reported in the Supplementary Materials.

### 2.7. Statistical Analysis

Continuous variables were summarised using mean and standard deviation or median and interquartile range according to distribution; categorical variables were reported as counts and percentages. Wilson 95% confidence intervals (CIs) were used for key patient-level proportions. For exploratory site comparisons, absolute prevalence differences were reported with Newcombe 95% CIs, together with Fisher’s exact tests. The six medication-derived site comparisons constituted one exploratory multiplicity family, for which Benjamini-Hochberg false-discovery-rate (FDR) adjusted q values were calculated. These comparisons remained hypothesis-generating irrespective of nominal *p*-values or FDR results.

For treatment ascertainment, a complete 2 × 2 table was constructed, and overall agreement, positive agreement, negative agreement, and Cohen’s kappa were calculated. Because repeated encounters were clustered within patients, 95% CIs for agreement measures were estimated using 10,000 nonparametric bootstrap replicates with resampling at the patient level (random seed 20260902). Sensitivity, specificity, positive predictive value, and negative predictive value were not estimated because neither routine data source constituted an independent clinical reference standard. No missing values were imputed; variable-specific denominators are reported where applicable. The study analysed the complete set of eligible records in the fixed study window, and no retrospective power calculation was used to reinterpret the available sample size. All tests were two-sided. Original analyses were performed in Python 3.13.5 using pandas 2.2.3, NumPy 2.3.5, SciPy 1.17.0, statsmodels 0.14.6, and Matplotlib 3.10.8. Reporting was guided by STROBE, RECORD, and ESMO-GROW recommendations [13,14,15]. Supporting analyses characterised monthly availability and agreement (Table S7), coding consistency and source overlap (Table S8), within-patient availability and consecutive-encounter transitions (Table S9), and structured data-quality checks (Table S10). An additional descriptive analysis examined the immediately preceding and following observed encounters for each single-agent medication-positive encounter within the same patient (Table S6). A different component meant a CRC-relevant medication class absent from the index encounter but present in either neighbour. All observed encounters were eligible as neighbours; oral prescriptions were excluded, and no maximum interval was imposed. Intervals were reported rather than interpreted as treatment cycles or clinical complementarity. A scenario analysis added hypothetical treatment-positive records among the 419 medication-unavailable, R001-negative encounters and recalculated agreement against this scenario-augmented indicator (Table S11). This scenario does not estimate missing treatment or validate either observed source. Alternative drug and procedure definitions and patient-level agreement are reported in Tables S3 and S5. Derived definitions are given in Table S12 and the cohort transformations in the Supplementary Methods transformation log.

### 2.8. Ethics and Data Protection

Direct identifiers were used only within the protected linkage/deduplication process and were removed from the analytical files after record linkage. Ethical approval No. 48/25.03.2026, dated 25 March 2026, was issued by the Comisia de Etică și Deontologie Universitară și Științifică, University of Medicine and Pharmacy of Craiova, for the project ‘Rectal cancer genetic and immunohistopathological correlations’. The investigators identify the present analysis of routine C18–C20 hospital data as part of this research project. Data abstraction began on 20 April 2026, after approval, and continued until eligible records through 30 April 2026 had been collected. Written informed consent for research participation and the use of medical data was obtained at admission; no consent waiver was used.

## 3. Results

### 3.1. Study Population and Documented Systemic Treatment Exposure

**Figure 1:**
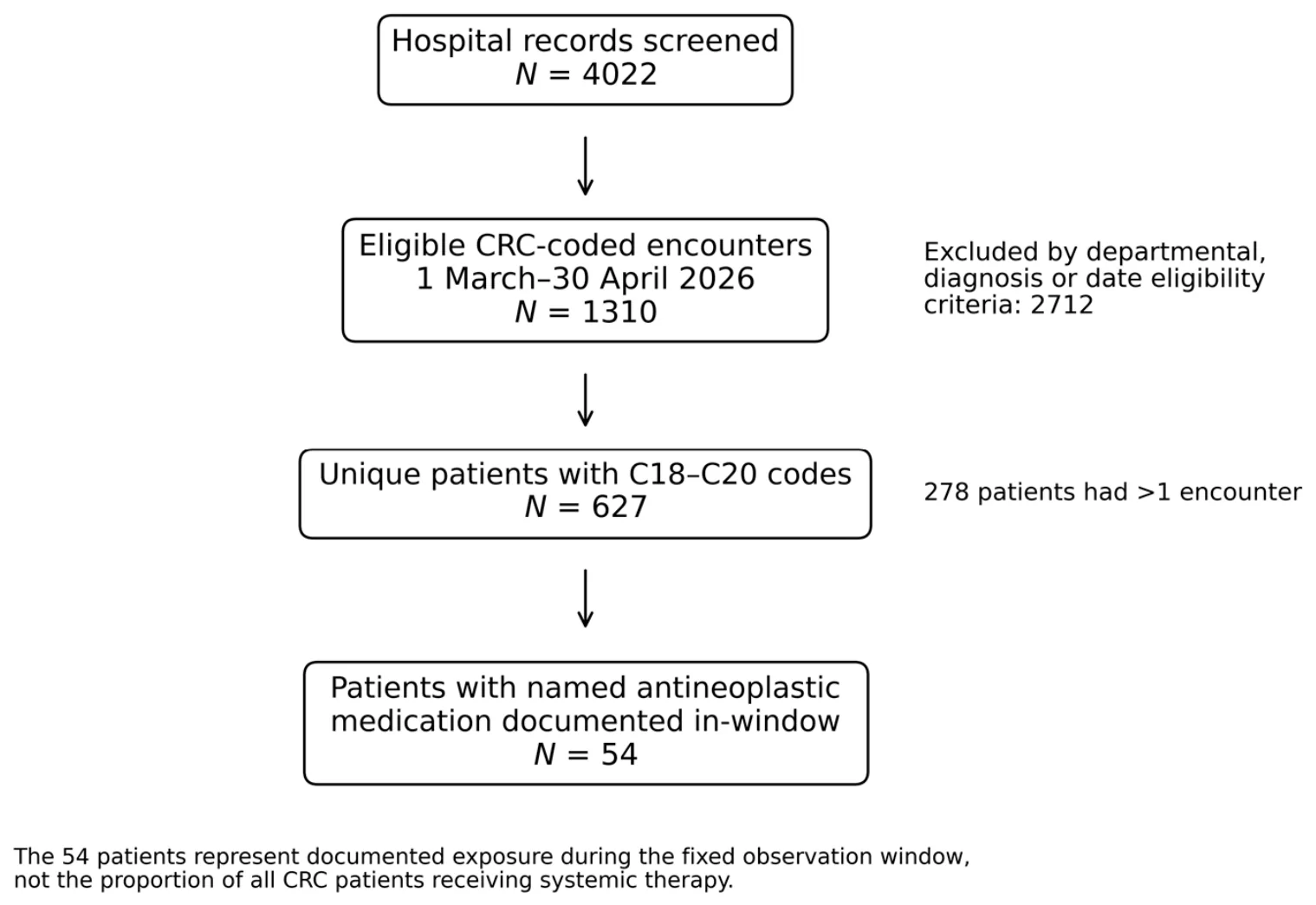
Study flow. The final 54-patient subgroup comprises patients with at least one named antineoplastic medication documented during the fixed study window; it is not an estimate of the overall proportion of CRC patients receiving systemic therapy. Exclusions reflect the combined eligibility criteria; separate counts for individual exclusion reasons were unavailable.

Of 4022 hospital records screened across departments and presentation types, 2712 did not meet the combined departmental, diagnosis, and date eligibility criteria, leaving 1310 CRC-coded encounters from 627 unique patients (Figure 1). There were 509 encounters in March and 801 in April; 1273 were day-hospital encounters, and 37 were continuous-admission encounters. The mean age at first observed encounter was 65.7 years (SD 10.0; *n* = 625; range 17–89), 386 patients (61.6%) were male, and 252 (40.2%) had rural residence. Patient-level site classification was C18 in 353 patients (56.3%), C19 in 32 (5.1%), and C20 in 242 (38.6%). Overall, 278 patients (44.3%) had more than one encounter during the study window.

A named antineoplastic medication was documented in at least one encounter for 54 patients (8.6% of the coded CRC population) and in 125 encounters. This proportion represents medication documentation during the fixed observation window, not the overall systemic-treatment rate. The 54-patient subgroup is selected by documentation availability and is not established as representative of the full CRC-coded population. Characteristics of the overall cohort and the treatment-documented subgroup are shown in Table 1.

### 3.2. Systemic Anticancer Treatment Landscape

Among the 54 patients in the treatment-documented subgroup, the two most frequent mutually exclusive medication patterns were cytotoxic component(s) only (20/54, 37.0%; 95% CI 25.4–50.4) and targeted/ICI plus cytotoxic components (20/54, 37.0%; 95% CI 25.4–50.4). Twelve patients (22.2%; 95% CI 13.2–34.9) had targeted/ICI components without cytotoxic co-documentation in the observed medication records, and two (3.7%; 95% CI 1.0–12.5) had other antineoplastic treatment only.

**Figure 2:**
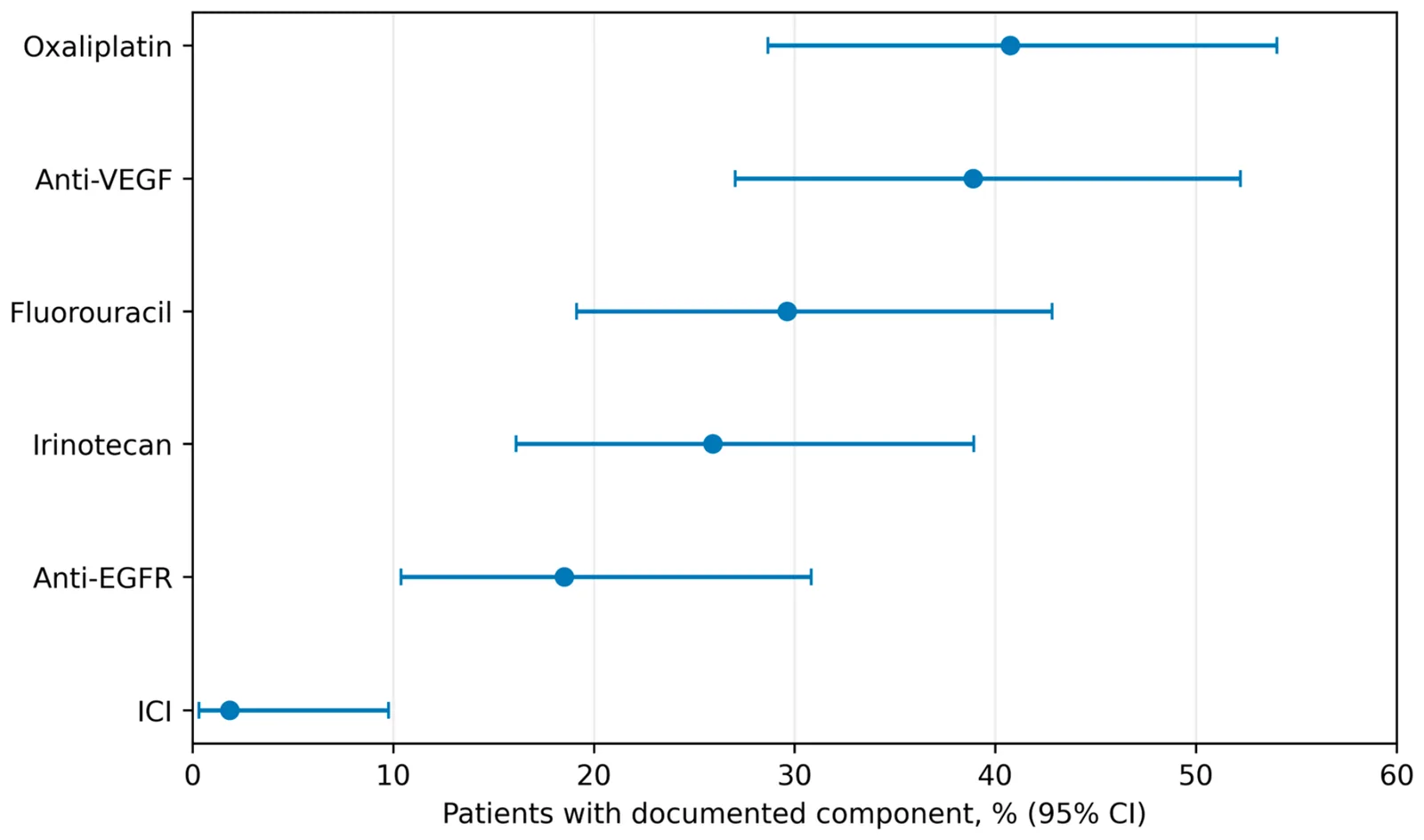
Medication-derived systemic anticancer treatment components among the 54 patients with at least one named antineoplastic medication documented during the study window. Error bars show Wilson 95% confidence intervals. Categories are not mutually exclusive. Oral prescription evidence is excluded from this figure and is reported separately.

At the medication-derived therapeutic-component level, oxaliplatin was documented in 22 patients (40.7%), anti-VEGF therapy in 21 (38.9%), fluorouracil in 16 (29.6%), irinotecan in 14 (25.9%), anti-EGFR therapy in 10 (18.5%), and an immune checkpoint inhibitor in 1 (1.9%). Fifty-two of 54 patients (96.3%) had at least one component from the CRC-relevant component set; the remaining two had only antineoplastic agents outside that set during the observation window. Oral prescriptions are reported separately in Section 3.5. The medication-derived treatment landscape is summarised in Table 2 and Figure 2.

### 3.3. Co-Documented Components and Repeated Treatment Documentation

**Figure 3:**
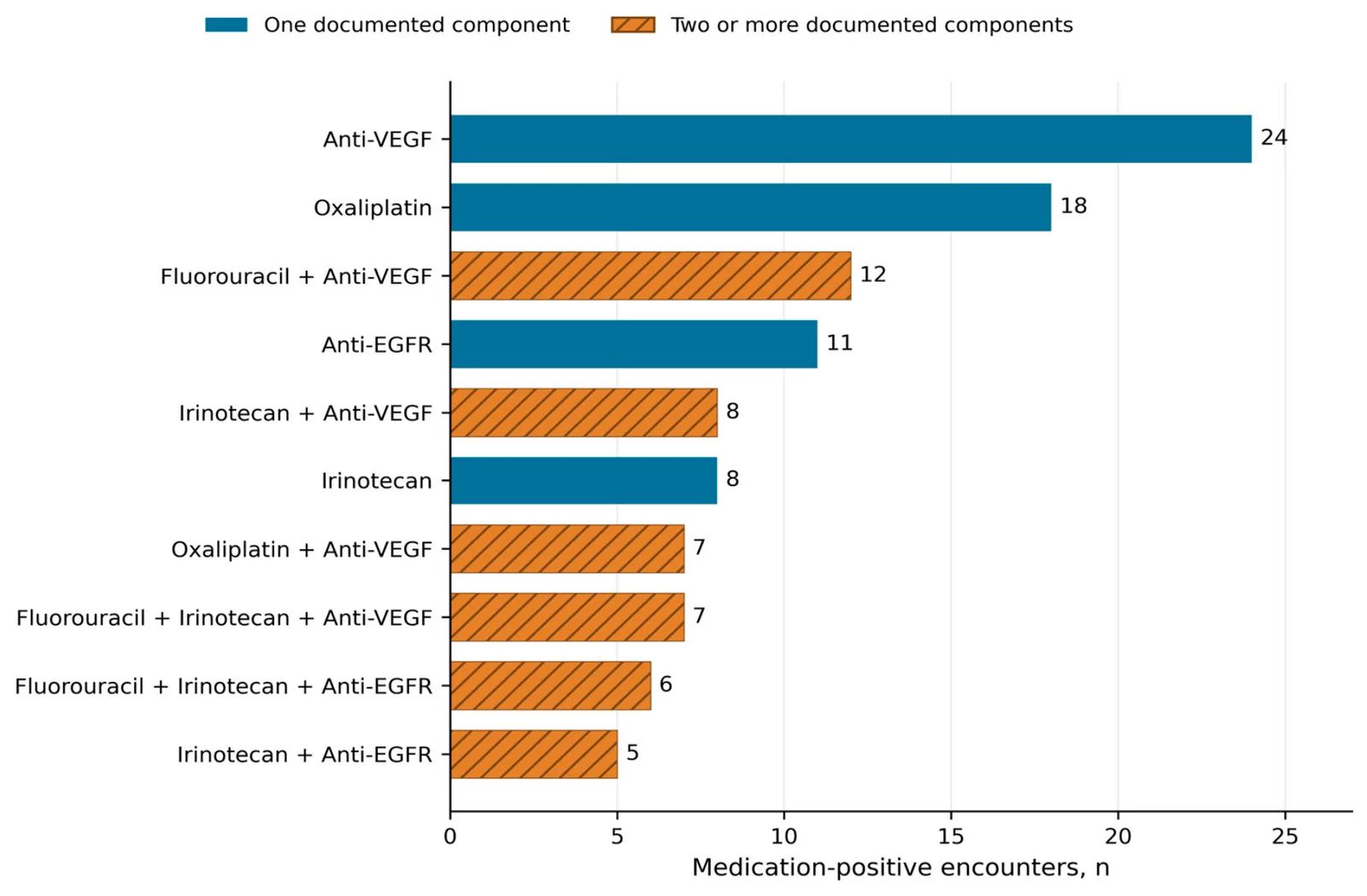
Most frequent exact same-encounter medication-derived therapeutic-component patterns among the 125 medication-positive encounters. The ten displayed patterns account for 106/125 encounters (84.8%) Colours distinguish one-component from multi-component patterns. Patterns are based on the medication field only; oral prescription evidence is analysed separately. Patterns are descriptive and are not assigned regimen names.

At the first treatment-documented encounter, 30 patients (55.6%) had one named antineoplastic agent, 16 (29.6%) had two, and 8 (14.8%) had three; thus, 24/54 (44.4%; 95% CI 32.0–57.6) had at least two named antineoplastic agents co-documented at that first observed treatment encounter. Across all 125 medication-positive encounters, 61 (48.8%; 95% CI 40.2–57.5) contained at least two named antineoplastic agents. Of the 64 single-agent encounters, two (3.1%), involving two patients, had a different CRC-relevant medication component in an immediately preceding or following encounter; one had fluorouracil in a neighbouring encounter. The three directional links were separated by 14, 14 and 20 days (Table S6). These observations do not identify split components of the same treatment cycle. The most frequent exact same-encounter medication-derived therapeutic-component patterns are shown in Figure 3.

Frequently co-documented same-encounter medication components included fluorouracil plus anti-VEGF (21 encounters; 7 patients), fluorouracil plus irinotecan (15 encounters; 7 patients), irinotecan plus anti-VEGF (15 encounters; 6 patients), fluorouracil plus anti-EGFR (12 encounters; 5 patients), irinotecan plus anti-EGFR (11 encounters; 3 patients), and fluorouracil plus oxaliplatin (6 encounters; 4 patients). These are descriptive co-occurrences within the medication field and were not assigned regimen names.

Twenty-nine patients (53.7%) had one distinct CRC-relevant component class documented across the study window, 15 (27.8%) had two, 7 (13.0%) had three, and 1 (1.9%) had four; two patients had no component in the CRC-relevant component set. Overall, 23/54 (42.6%; 95% CI 30.3–55.8) therefore had at least two medication-derived CRC-relevant therapeutic classes documented. Thirty-three patients (61.1%) had medication-positive treatment documentation in at least two encounters. Among these 33 patients, 30 (90.9%; 95% CI 76.4–96.9) had an identical documented class set across all observed treatment encounters; the remaining three had a changed documented class set. This stability measure describes repeated documentation only and does not establish treatment-cycle continuity or regimen persistence.

### 3.4. Exploratory Treatment Patterns by Colorectal Tumour-Site Group

Among the 54 patients with documented antineoplastic exposure, 32 were classified as C18 and 22 as C19–C20. Most medication-derived component frequencies were similar between groups (Table 3). The largest exploratory contrast concerned anti-EGFR therapy, documented in 2/32 C18 patients (6.3%) and 8/22 C19–C20 patients (36.4%), corresponding to an absolute difference of 30.1 percentage points (95% CI 8.4–51.3; Fisher *p* = 0.010). Across the six medication-derived site comparisons, the Benjamini-Hochberg FDR q value for anti-EGFR was 0.060.

The anti-EGFR contrast remained similar in a sensitivity analysis excluding the three treatment-documented patients with inconsistent C18–C20 site codes across encounters (2/31 vs. 8/20; absolute difference 33.5 percentage points, 95% CI 10.5–55.4; Fisher *p* = 0.008). These findings are hypothesis-generating because C18 versus C19–C20 does not represent biological sidedness and the dataset did not contain RAS/BRAF status, metastatic status, or treatment line. No comparison met a Benjamini-Hochberg FDR threshold of q < 0.05; these data do not support a clinical site-specific treatment recommendation.

### 3.5. Oral Treatment Documentation

Seven encounters from seven patients had an antineoplastic oral prescription recorded during the study window: four for capecitabine and three for trifluridine-tipiracil; regorafenib was not identified. Prescription evidence was analysed separately from medication-derived treatment patterns because a prescription does not demonstrate administration. All seven patients were already identified as treatment-documented by the medication field; six of these seven prescription-positive encounters also carried the R001 procedure indicator. Thus, the prescription field added context for oral therapy but did not add new treatment-documented patients in this study window.

### 3.6. Concordance of Routine Data Sources for Treatment Ascertainment

Medication information was nonblank in 891/1310 encounters (68.0%) and unavailable in 419 (32.0%). Among the 891 interpretable encounters, 124 were positive by both the R001 procedure indicator and named antineoplastic medication, 766 were negative by both sources, one was medication-positive/procedure-negative, and none was procedure-positive/medication-negative (Table 4). Overall agreement was 99.89% (95% patient-cluster bootstrap CI 99.64–100.00), positive agreement was 99.60% (98.59–100.00), negative agreement was 99.93% (99.79–100.00), and Cohen’s kappa was 0.9953 (95% CI 0.9839–1.0000). The single medication-positive/procedure-negative encounter contained bevacizumab in the medication field and trifluridine-tipiracil in the prescription field (Table S8). With only one discordant encounter, many patient-cluster bootstrap resamples contain no discordance; the upper CI boundary of 1.0000 does not establish perfect agreement in the underlying population. The exact McNemar result is retained in Table S3 as a supporting statistic.

None of the 419 medication-unavailable encounters contained an R001 procedure indicator. Their joint lack of procedure and medication evidence does not establish treatment absence and remains compatible with shared under-capture. Full-cohort operational and alternative-definition sensitivity analyses are reported in the Supplementary Materials. Medication availability was 324/509 (63.7%) in March and 567/801 (70.8%) in April (Table S7). Among 278 patients with repeated encounters, 148 (53.2%) had medication information at every encounter, 103 (37.1%) had mixed availability, and 27 (9.7%) had none (Table S9). In the hypothetical scenario, 11 hidden positives among 419 blank records (2.6%) reduced kappa below 0.95 (to 0.949; Table S11); this illustrates sensitivity to assumptions, not the number of treatments actually missed. The available audit also identified 18 patients with inconsistent site codes and four encounters involving unexpected agents (Tables S8 and S10).

## 4. Discussion

### 4.1. Principal Findings

In this CRC-specific real-world study, the main finding was a structured picture of medication-documented systemic treatment components during routine care rather than a single drug frequency. Among 54 patients with named antineoplastic medication documented during the fixed two-month window, 37.0% had cytotoxic components only, 37.0% had targeted/ICI plus cytotoxic components, 22.2% had targeted/ICI components without cytotoxic co-documentation, and 3.7% had only other antineoplastic agents. Nearly half had at least two named antineoplastic agents at their first treatment-documented encounter, 42.6% had at least two medication-derived CRC-relevant therapeutic classes documented across the window, and 61.1% had treatment documented repeatedly. Among patients with repeated treatment documentation, documented class sets were unchanged in 90.9%.

### 4.2. Clinical Context of the Documented CRC Treatment Landscape

The observed components are clinically recognisable within contemporary CRC systemic treatment strategies. In addition to current ESMO guidance [2], diverse real-world cohorts from North America and Europe consistently report fluoropyrimidine/oxaliplatin- and irinotecan-based treatment backbones, with bevacizumab, anti-EGFR therapy, and other targeted or immune approaches variably incorporated according to disease biology, line of therapy, and practice setting [7,16,17,18,19,20,21,22,23,24]. Stage- and site-specific guidelines for colon and rectal cancer further emphasise that systemic therapy must be interpreted within a broader multidisciplinary context [3,4]. Our medication data similarly showed substantial documentation of oxaliplatin, fluorouracil, irinotecan, bevacizumab, and anti-EGFR therapy, while capecitabine and trifluridine-tipiracil appeared in the separate prescription field. Direct comparison of percentages with metastatic or line-specific cohorts would be inappropriate because our population was defined by C18–C20 diagnosis codes, included an unknown mixture of disease stages and treatment indications, and was observed for only two months.

Within Romania, real-world evidence has also documented routine use of bevacizumab-containing systemic therapy in metastatic CRC [19]. The co-documentation analysis describes recorded combinations of components. Fluorouracil plus oxaliplatin, fluorouracil plus irinotecan, and medication combinations involving anti-VEGF or anti-EGFR therapy are recognisable building blocks of CRC systemic treatment [2,3,4,16,17,18,19,20,21,22,23,24]. Nevertheless, we deliberately described these as co-documented therapeutic components rather than assigning regimen names. Routine medication fields do not, by themselves, establish protocol intent, dose, cycle, or treatment line, and recent methodological work has highlighted the importance of explicit exposure definitions in CRC RWE [8]. Apparent single-component records may reflect incomplete capture of a partner drug, including infusional fluorouracil, or components recorded at different encounters. The dictionary includes both named fluorouracil products and an elastomeric infusion-pump device; the device was not treated as evidence of fluorouracil administration. Medication was recorded in a single hospital field, but independent pharmacy or administration validation was unavailable. The adjacent-encounter analysis found a different component for only two single-agent records, at intervals of 14–20 days; it cannot establish whether omitted or separately recorded components belonged to the same cycle. The reported categories should not be read as monotherapy frequencies.

Interpretation also depends on clinical information beyond medication names. Work on locally advanced CRC [25] and sentinel-node assessment [26] addresses disease extent and pathological staging, which were unavailable here. A review of T4 colon cancer management further considers staging, resectability and perioperative treatment [27]. These clinical dimensions cannot be reconstructed from the recorded medication entries alone.

Hospital contacts may also concern investigation or management of postoperative complications. A retrospective study of rectal cancer surgery describes the diagnosis and management of such complications [28], providing CRC-specific context for distinguishing hospital contact from systemic treatment delivery. Individual postoperative complications and reasons for admission were not systematically classified in the present cohort.

### 4.3. Repeated Documentation and Treatment-Pattern Complexity

The derived measures of first treatment-documented encounter complexity and repeated documentation of the same class set were introduced to describe information already present in the fixed study window while avoiding unsupported longitudinal inference. The fact that 44.4% of patients had at least two named antineoplastic agents co-documented at the first observed treatment encounter indicates that multi-component treatment documentation was common. Likewise, the frequent recurrence of the same documented class set among patients with repeated treatment encounters suggests that the observed patterns were often reproduced across more than one contact. These measures should not be interpreted as treatment cycles, regimen persistence, or duration because treatment before and after the observation window was not evaluated. If components from one cycle are split across encounters, the documented set may appear to change; repeated omission of the same partner drug may instead make it appear unchanged. Components before March or after April are outside observation, so neither result identifies clinical continuity or switching.

### 4.4. Exploratory Comparison by Coded Anatomical Site

Anti-EGFR therapy was more frequently documented in the C19–C20 group than in the C18 group, with an observed absolute difference that remained after excluding patients with inconsistent site coding. This descriptive contrast did not meet an FDR threshold of q < 0.05 and does not establish a biological site effect. C18 versus C19–C20 does not reproduce biological right- versus left-sided CRC, particularly because most C18 codes did not contain sufficiently granular subsite information. Moreover, RAS/BRAF status, metastatic status, tumour sidedness, and treatment line are central to interpreting anti-EGFR use in contemporary metastatic CRC care [2]. The FDR-adjusted q value of 0.060 further supports restrained interpretation rather than a confirmatory claim.

### 4.5. Oral Therapy and Data-Source Complementarity

Oral treatment is a recognised challenge for routine data ascertainment. In Medicare data, capecitabine was less sensitively captured than intravenously administered CRC agents when compared with a clinical reference source [11], and a national English colon-cancer study found that combining chemotherapy and administrative datasets reduced under-capture, particularly for oral treatment [10]. In our study, oral prescriptions were uncommon and did not identify additional treatment-documented patients beyond the medication source. Even so, retaining prescription information as a separate complementary domain was methodologically preferable to assuming that oral exposure would be represented in the same way as infusion therapy.

### 4.6. Treatment Ascertainment and Reproducibility

The near-complete same-encounter concordance describes agreement between two fields in the same record; it does not establish completeness of the documented treatment components. At the same time, high concordance should not be equated with clinical accuracy. The procedure and medication domains arose within the same hospital workflow and may share upstream documentation or billing processes, so their errors need not be independent. CRC-specific studies that compared administrative algorithms with chart-based or manually abstracted sources have shown that ascertainment performance depends strongly on coding rules, data source, and clinical construct [29,30]. Agreement reporting itself requires explicit definition of the construct, units, denominators, and statistical methods [31], while broader evaluations of routinely collected health-data studies have shown that coding algorithms, linkage, missingness, and reproducibility are often incompletely reported [32]. These concerns are consistent with general principles for real-world evidence, which emphasise that data provenance and fitness for the research question are fundamental to interpretation [33]. Our result is therefore best interpreted as internal consistency of treatment ascertainment within the studied data environment, not external validation.

One methodological strength of the present analysis is the explicit construction of treatment exposure. Recent CRC-specific evidence shows that drug-exposure definitions are often incompletely reported in RWE studies [8]. We therefore retained the raw-token-to-agent dictionary, kept medication-derived treatment patterns separate from prescription evidence, distinguished same-encounter co-documentation from patient-level ever documentation, excluded leucovorin from antineoplastic counts, and specified the interpretation of each derived variable. This level of transparency directly addresses reproducibility concerns highlighted for routinely collected health data [32,33] and supports interpretation even though the underlying source is less structured than dedicated systemic anticancer therapy datasets [9].

### 4.7. Strengths and Limitations

Strengths include assembly of the cohort from patient records, national-identifier-based patient linkage, removal of direct identifiers after linkage, preservation of repeated encounters, a complete raw-token medication dictionary, clearly defined patient- and encounter-level treatment constructs, effect-size reporting for exploratory site comparisons, multiplicity awareness, patient-cluster bootstrap confidence intervals for encounter-level agreement, and sensitivity analyses that did not recode unavailable medication fields as clinical treatment absence in the primary analysis.

Several limitations are substantial. First, the study reflects a single local hospital data environment and a fixed two-month observation window; it cannot describe complete treatment histories or population treatment rates. Second, only 54 patients had named antineoplastic medication documented during the window, limiting precision for subgroup comparisons and precluding complex multivariable modelling. Third, the treatment-pattern research question was developed after an initial audit of the available data; the treatment-pattern analyses are therefore descriptive and exploratory, without a claim of prospective pre-specification. Fourth, the dataset lacked sufficiently complete tumour stage, histopathology, molecular biomarkers, treatment intent, and treatment line, all of which are central to clinical interpretation of CRC treatment. Fifth, C18–C20 diagnosis codes were not treated as histopathological confirmation, and the exploratory C18 versus C19–C20 comparison cannot substitute for biological sidedness; the observed anti-EGFR difference cannot be interpreted without RAS status, metastatic status, and treatment line. Sixth, the medication token-to-agent dictionary was rule-based and reviewed by an oncologist, but was not independently validated against pharmacy or medication-administration data. Seventh, prescription evidence was retained as a separate complementary domain and does not prove oral drug administration. Eighth, procedure and medication indicators were not independent reference standards and may share upstream documentation processes. Ninth, medication information was unavailable in 32.0% of encounters, although none of those encounters carried the primary R001 procedure indicator. Finally, findings from a single data environment may not generalise to other hospitals, coding systems, or national healthcare infrastructures. Admission-diagnosis eligibility may omit CRC encounters coded primarily for chemotherapy or metastatic disease. Department-specific proportions, reasons for individual admissions, and complete cross-centre treatment histories were unavailable. Drug indication was not clinically verified for the two patients with unexpected agents: carboplatin plus etoposide in three encounters from one patient and gemcitabine in one encounter from another. The findings cannot establish that all identified antineoplastic medication was given for CRC.

## 5. Conclusions

During a fixed two-month period of routine hospital care, patients with coded colorectal cancer showed heterogeneous patterns of medication-documented systemic anticancer treatment, including cytotoxic, targeted, and immune components, while oral prescriptions provided complementary information. Multi-component and repeated treatment documentation were common among patients with observed antineoplastic medication, and the same documented class set often recurred across observed encounters.

Procedure- and medication-based indicators showed near-complete concordance, describing internal consistency within a shared hospital workflow while not excluding joint under-capture or establishing clinical accuracy, treatment appropriateness, or effectiveness. These findings support carefully defined use of routine hospital data for descriptive CRC treatment-pattern research, with explicit recognition of the limitations imposed by short observation, incomplete clinical context, and non-independent data sources.

## Figures and Tables

**Table 1:** Characteristics of the colorectal cancer study population.

Characteristic	Overall Cohort (*N* = 627)	Treatment-Documented Subgroup (*N* = 54)
Age at first observed encounter, years, mean (SD)	65.7 (10.0), *n* = 625	67.7 (8.9)
Male sex	386 (61.6%)	28 (51.9%)
Female sex	241 (38.4%)	26 (48.1%)
Urban residence	375 (59.8%)	37 (68.5%)
Rural residence	252 (40.2%)	17 (31.5%)
C18 site group	353 (56.3%)	32 (59.3%)
C19 site group	32 (5.1%)	4 (7.4%)
C20 site group	242 (38.6%)	18 (33.3%)
Observed encounters per patient, median (IQR)	1 (1–2)	3 (2–5)
>1 encounter during study window	278 (44.3%)	47 (87.0%)
Any continuous-admission encounter	20 (3.2%)	14 (25.9%)

The treatment-documented subgroup is nested within the overall cohort. No inferential comparison between the columns was undertaken because documentation within a two-month window does not define a clinical treated-versus-untreated contrast. IQR, interquartile range; SD, standard deviation.

**Table 2:** Documented systemic anticancer treatment patterns among patients with named antineoplastic medication exposure (*N* = 54).

Measure	Patients, *n*	%	95% CI
**A. Mutually exclusive medication-derived patterns**			
Cytotoxic component(s) only	20	37.0	25.4–50.4
Targeted/ICI + cytotoxic component(s)	20	37.0	25.4–50.4
Targeted/ICI without cytotoxic co-documentation	12	22.2	13.2–34.9
Other antineoplastic only	2	3.7	1.0–12.5
B. Medication-derived therapeutic components *			
Oxaliplatin	22	40.7	28.7–54.0
Anti-VEGF (bevacizumab)	21	38.9	27.0–52.2
Fluorouracil	16	29.6	19.1–42.8
Irinotecan	14	25.9	16.1–38.9
Anti-EGFR (cetuximab/panitumumab)	10	18.5	10.4–30.8
Immune checkpoint inhibitor	1	1.9	0.3–9.8
**C. Complementary oral-prescription documentation**			
Any oral antineoplastic prescription	7	13.0	6.4–24.4
Capecitabine prescription	4	7.4	2.9–17.6
Trifluridine-tipiracil prescription	3	5.6	1.9–15.1
**D. Medication-derived complexity/repeated-documentation measures**			
≥2 named antineoplastic agents at first treatment-documented encounter	24	44.4	32.0–57.6
≥2 CRC-relevant components documented during the window	23	42.6	30.3–55.8
≥2 medication-positive treatment encounters	33	61.1	47.8–73.0
Unchanged documented class set among patients with repeated documentation	30	90.9 **	76.4–96.9

* Medication-derived component categories are not mutually exclusive and are based on the encounter medication field unless explicitly labelled as prescription evidence. The mutually exclusive patterns and complexity measures are medication-derived. Oral prescriptions are displayed separately and are not treated as proof of administration. ** Denominator = 33 patients with at least two medication-positive treatment encounters. ICI, immune checkpoint inhibitor; VEGF, vascular endothelial growth factor; EGFR, epidermal growth factor receptor.

**Table 3:** Exploratory documented treatment components by colorectal tumour-site group among treatment-documented patients.

Component	C18 (*n* = 32)	C19-C20 (*n* = 22)	Absolute Difference, pp (95% CI) *	Fisher p
Fluorouracil (medication field)	10/32 (31.3%)	6/22 (27.3%)	−4.0 (−26.3 to +20.8)	1.000
Oxaliplatin	13/32 (40.6%)	9/22 (40.9%)	+0.3 (−24.3 to +25.6)	1.000
Irinotecan	7/32 (21.9%)	7/22 (31.8%)	+9.9 (−12.9 to +33.5)	0.531
Anti-VEGF	13/32 (40.6%)	8/22 (36.4%)	−4.3 (−28.1 to +21.4)	0.784
Anti-EGFR	2/32 (6.3%)	8/22 (36.4%)	+30.1 (+8.4 to +51.3)	0.010
Immune checkpoint inhibitor	1/32 (3.1%)	0/22 (0.0%)	−3.1 (−15.7 to +12.0)	1.000

* Difference calculated as C19-C20 minus C18; 95% confidence intervals use the Newcombe method. The six medication-derived component comparisons formed one exploratory multiplicity family. The FDR-adjusted q value for the anti-EGFR comparison was 0.060; all other q values were 1.000. These comparisons were exploratory. pp, percentage points.

**Table 4:** Primary encounter-level concordance of procedure- and medication-based treatment indicators.

Measure	Estimate/Count	95% CI	Interpretation
Both sources positive	124	-	R001 positive and named antineoplastic documented
Procedure positive only	0	-	No named antineoplastic in nonblank medication field
Medication positive only	1	-	Named antineoplastic documented without R001
Both sources negative	766	-	Neither indicator positive
Overall agreement	99.89%	99.64–100.00	Patient-cluster bootstrap CI
Positive agreement	99.60%	98.59–100.00	Symmetric positive agreement
Negative agreement	99.93%	99.79–100.00	Symmetric negative agreement
Cohen’s kappa	0.9953	0.9839–1.0000	Chance-corrected agreement; not clinical validity
Exact McNemar p	1.000	-	Exact two-sided paired test
Medication-unavailable encounters with R001	0/419	-	Blank medication field remains unavailable, not treatment-negative

## Data Availability

Aggregate results and the complete medication dictionary are provided in the article and Supplementary Materials. Patient-level data and analysis code are not included in these materials.

## Supplementary Materials

The following supporting information can be downloaded at: https://www.mdpi.com/article/10.3390/jcm15218486/s1, Table S1, local procedure-code implementation and analytical roles; Table S2, complete raw medication token-to-normalised agent dictionary; Table S3, complete agreement metrics with patient-cluster bootstrap confidence intervals; Table S4, missing-data and completeness audit; Table S5, alternative-definition and operational full-cohort sensitivity analyses; Table S6, descriptive adjacent-encounter medication analysis; Tables S7–S10, temporal, consistency, source-complementarity, and within-patient documentation summaries; Table S11, tipping-point sensitivity analysis; Table S12, derived-variable definitions and interpretation limits; Supplementary Methods, cohort reconstruction, deduplication, medication normalisation, and reproducibility details. The supplementary file includes the medication dictionary and analytical definitions; no separate code archive or reporting checklists are included in the present revision package.

## Abbreviations

The following abbreviations are used in this manuscript:

CI	confidence interval
CRC	colorectal cancer
EGFR	epidermal growth factor receptor
FDR	false discovery rate
ICI	immune checkpoint inhibitor
IQR	interquartile range
RECORD	Reporting of studies conducted using Observational Routinely-collected health Data
RWD	real-world data
SD	standard deviation
STROBE	Strengthening the Reporting of Observational Studies in Epidemiology
VEGF	vascular endothelial growth factor
ESMO-GROW	ESMO Guidance for Reporting Oncology real-World evidence
BRAF	B-Raf proto-oncogene
RAS	rat sarcoma proto-oncogene family
RWE	real-world evidence
SACT	systemic anti-cancer therapy
R001	Romanian RO DRG chemotherapy-administration procedure-code family
