# Supplementary material for: Medication-Documented Systemic Anticancer Treatment in Colorectal Cancer: A Single-Centre Routine-Data Study
Source: J Clin Med. 2026 Oct 31;15(21):8486. doi: 10.3390/jcm15218486 (PMC13764127; doi:10.3390/jcm15218486)
Supplement: Supplementary file 1 [file jcm-15-08486-s001.zip › jcm-4588226-supplementary.pdf]

Supplementary Materials

Supplementary Materials for: Medication-Documented Systemic Anticancer Treatment in Colorectal Cancer: A Single-Centre Routine-Data Study

This supplementary file corresponds to the revised manuscript and contains technical implementation, reproducibility, missingness, sensitivity, and supporting agreement analyses. Direct identifiers are not included.

Supplementary Methods: Cohort Reconstruction, Linkage and Medication Parsing

Information was abstracted manually from patient records into Excel. The screening source comprised 4,022 hospital records across departments and presentation types. Eligibility required an oncology, internal medicine, or surgery encounter, a C18-C20 admission diagnosis, and a date from 1 March 2026 to before 1 May 2026. Applying these combined criteria retained 1,310 encounters and excluded 2,712 records; separate counts by exclusion reason were unavailable. The two-month window reflected access to the study data.

Patient linkage used the national identifier (CNP) as the primary key before data cleaning and removal of direct identifiers. Sequential study and encounter identifiers were assigned after linkage. The final analytical data comprised 627 patients and 1,310 encounters; direct identifiers were not included.

Age and sex were derived internally from the national identifier where the century component was interpretable. Two ages were left missing rather than inferred. Direct identifiers were not exported. The study team verified the recorded age of 17 years as correct.

Procedure tokens were taken from proc Sec because procprinc was blank in all 1,310 included encounters. Tokens were split on commas, semicolons, or line breaks and deduplicated within each encounter. The main manuscript uses functional procedure families (administration-type R001 evidence versus planning/service-context evidence) to preserve international interpretability; exact local codes and their documentary sources are retained here for reproducibility.

The medication field was split on semicolons. Empty tokens were removed. Two standalone '-1' quantity fragments generated by a semicolon before the suffix were merged with the preceding product token. The resulting 5,075 token occurrences represented 186 distinct raw token strings.

Medication classification was rule-based and reviewed by an oncologist. Antineoplastic positivity required an identifiable antineoplastic active agent. Calcium folinate (leucovorin) was classified as a treatment modulator, not an antineoplastic. Supportive therapies and fluids were excluded from the antineoplastic indicator. Medication was recorded in a single hospital medication field; no independent validation against pharmacy or medication-administration records was performed.

For the CRC-centred manuscript, patient-level medication patterns and medication-derived class counts use the medication field only. The separate prescription field is retained as complementary evidence for oral antineoplastic therapy and is not treated as proof of administration. Any sensitivity analysis that combines medication and prescription information is labelled explicitly.

Blank medication fields were coded as unavailable. They were not coded as treatment-negative in the primary analysis. An operational full-cohort sensitivity scenario coded blanks negative solely to quantify the impact of that assumption.

Table S1. Procedure-code definitions and analytical roles.

Code	Encounters	Patients	Med available	Med blank	Analytical role	Nomenclature description
**R00101**	89	34	89	0	Primary R001 component	R00101: intravenous chemotherapy lasting up to 1 hour or duration unspecified.
**R00102**	75	38	75	0	Primary R001 component	R00102: intravenous chemotherapy lasting over 1 and up to 6 hours.
**R00103**	25	12	25	0	Primary R001 component	R00103: intravenous chemotherapy lasting over 6 hours.
**R00107**	2	2	2	0	Primary R001 component	R00107: oral chemotherapy.
**R00108**	0	0	0	0	Primary R001 component	R00108: subcutaneous or intramuscular chemotherapy.
**R00301**	2	2	2	0	Broad sensitivity only	R00301: planning of a primary chemotherapy course.
**R00302**	33	24	30	3	Broad sensitivity only	R00302: planning of a subsequent chemotherapy course.
**SPT_3.7**	98	40	98	0	Broad sensitivity only	Service-context code; an authoritative label for SPT_3.7 was not established in this revision.

R00108 was included in the selected procedure indicator but was not observed. The indicator uses R00101, R00102, R00103, R00107, and R00108 rather than every code in the R001 family. R001 denotes chemotherapy administration in the RO DRG v1 procedure classification. Code descriptions above are English translations of the official classification, Chapter XVIII, printed page 260 (PDF page 262): https://drg.inmss.ro/DocDRG/download.php?fi=26 (accessed 17 October 2026). Concordance does not confirm the completeness or accuracy of local coding.

Table S2. Complete raw medication token to normalised agent dictionary.

Raw token	Normalised agent	Category	Antineoplastic	CRC-related / treatment-related	Ambiguous	Token n	Encounter n	Patient n
**SOLUTIE RINGER 500 ML BRAUN-1**	Ringer solution	fluid_electrolyte	0	0	0	398	363	210
**DEXAMETHASONE SODIUM PHOSPHATE 8 mg/2 ml-1**	dexamethasone	supportive	0	0	0	374	342	174
**VITAMINA B1 100MG/2ML-1**	thiamine (vitamin B1)	supportive	0	0	0	362	342	235
**VITAMINA B6 250MG/ML-1**	pyridoxine (vitamin B6)	supportive	0	0	0	362	342	235
**VITAMINA C 500 mg/5ml-2**	ascorbic acid (vitamin C)	supportive	0	0	0	347	336	238
**OMEPRAZOL ROMPHARM 40 MG-1**	omeprazole	supportive	0	0	0	220	155	82
**ALGOCALMIN 1G/2ML*5F-1**	metamizole	supportive	0	0	0	206	171	105
**NO-SPA 40 mg/2 ml-1**	drotaverine	supportive	0	0	0	200	167	91
**CLORURA DE SODIU 0.9% 250 ml-1**	sodium chloride solution	fluid_electrolyte	0	0	0	153	125	74
**OMEZ 40 mg-1**	omeprazole	supportive	0	0	0	134	133	84
**METAMIZOL KALCEKS 500MG/ML *2ML-1**	metamizole	supportive	0	0	0	127	117	98
**CLORURA DE SODIU 9 mg/500ML BRAUN-1**	sodium chloride solution	fluid_electrolyte	0	0	0	120	110	72
**PANTOPRAZOL ROMPHARM 40 MG-1**	pantoprazole	supportive	0	0	0	119	109	57

**SOLUTIE RINGER 500ML* 10FL-1**	Ringer solution	fluid_electrolyte	0	0	0	117	117	94
**GLUCOZA 5% 500 ML STADA-1**	glucose solution	fluid_electrolyte	0	0	0	112	102	88
**CLORURA DE SODIU 0,9% 100ML BRAUN-1**	sodium chloride solution	fluid_electrolyte	0	0	0	97	80	55
**DEXKETOPROFEN ROMPHARM 50 mg/2 ml-1**	dexketoprofen	supportive	0	0	0	84	75	44
**GLUCOSE 5%-500ML BRAUN-1**	glucose solution	fluid_electrolyte	0	0	0	77	69	67
**GRANISETRON KABI 1mg/ml 3MG/3ML-1**	granisetron	supportive	0	0	0	59	55	35
**GLUCOZA 5% 250mlx30pg+2tub-1**	glucose solution	fluid_electrolyte	0	0	0	55	47	30
**CLORURA DE SODIU 0,9%-250 ml Braun-1**	sodium chloride solution	fluid_electrolyte	0	0	0	53	45	42
**HIDROCORTIZON HF 100 mg-HF-1**	hydrocortisone	supportive	0	0	0	50	46	33
**CLORURA DE SODIU 9 mg/500ML BRAUN-2**	sodium chloride solution	fluid_electrolyte	0	0	0	47	38	14
**METOCLOPRAMID TERAPIA 5mg/ml* 2 ML-1**	metoclopramide	supportive	0	0	0	46	41	30
**FOLINAT DE CALCIU KALCEKS 100 MG/10 ML-4**	calcium folinate (leucovorin)	treatment_modulator	0	1	0	39	26	11
**ASPATOFORT 10 ML-2**	Aspatofort (trade name)	supportive	0	0	1	38	31	20
**DEXAMETHASONE SODIUM PHOSPHATE 8 mg/2 ml-2**	dexamethasone	supportive	0	0	0	37	25	17
**KETOTIFEN LPH 1 mg-1**	ketotifen	supportive	0	0	0	36	30	23
**THIOSSEN TURBO 600MG/50ML-1**	Thiossen Turbo (trade name)	supportive	0	0	1	34	34	15
**OYAVAS 400 mg/16 ml-1**	bevacizumab	antineoplastic_targeted	1	1	0	31	31	14
**HEPARINA GALENIKA 5000 UI/ml-1**	heparin	supportive	0	0	0	30	25	18
**POMPA ELASTOMERICA 275ML(5ML/H)-1**	elastomeric infusion pump	device	0	0	0	30	25	11
**CLORURA DE SODIU 0.9% 250 ml-2**	sodium chloride solution	fluid_electrolyte	0	0	0	29	24	14
**SULFAT DE ATROPINA TAKEDA 1 mg/ml* 5 fiole* 1 ml-1**	atropine	supportive	0	0	0	29	27	14
**OSETRON 2MG/ML 8MG/4ML-1**	Osetron (antiemetic trade name)	supportive	0	0	1	27	25	20
**PANTOPRAZOL SUN 40MG-1**	pantoprazole	supportive	0	0	0	27	24	20
**PARACETAMOL KABI 10 mg/ml*100 ML-1**	paracetamol	supportive	0	0	0	27	27	24
**DERMODRIN 30 mg/2 ml* 5 FIOLE-1**	Dermodrin (trade name)	supportive	0	0	1	26	17	14
**ARGININA SORBITOL*250ML-1**	arginine/sorbitol solution	supportive	0	0	0	25	23	14
**OXALIPLATIN KABI 100MG 5 mg/ml-1**	oxaliplatin	antineoplastic_cytotoxic	1	1	0	24	15	14
**OXALIPLATIN KABI 50 MG 5 mg/ml-1**	oxaliplatin	antineoplastic_cytotoxic	1	1	0	19	10	9
**5 FLUOROURACIL EBEWE 1000MG 50MG/ML-2**	fluorouracil	antineoplastic_cytotoxic	1	1	0	15	9	3
**FLUIMUCIL 300MG/3ML-2**	acetylcysteine	supportive	0	0	0	15	13	12
**ALBUMINA UMANA BAXALTA 200 g/l 100 ml-1**	human albumin	supportive	0	0	0	14	12	10

**CLORURA DE SODIU 0.9% 250 ml-3**	sodium chloride solution	fluid_electrolyte	0	0	0	14	12	8
**OXALIPLATIN ACCORD 100MG/20ML-1**	oxaliplatin	antineoplastic_cytotoxic	1	1	0	14	12	11
**VENOFER 100mg fer/5ml-2**	iron sucrose	supportive	0	0	0	14	12	7
**5 FLUOROURACIL EBEWE 1000MG 50MG/ML-4**	fluorouracil	antineoplastic_cytotoxic	1	1	0	13	10	5
**IRINOTECAN ACCORD 300 mg/15ml-1**	irinotecan	antineoplastic_cytotoxic	1	1	0	13	11	5
**ERBITUX 100mg/20ml-8**	cetuximab	antineoplastic_targeted	1	1	0	12	10	4
**KETOTIFEN LAROPHARM 1 mg-1**	ketotifen	supportive	0	0	0	12	10	10
**VITAMINA C 500 mg/5ml-3**	ascorbic acid (vitamin C)	supportive	0	0	0	12	12	6
**5 FLUOROURACIL EBEWE 1000MG 50MG/ML-1**	fluorouracil	antineoplastic_cytotoxic	1	1	0	11	6	3
**ALBUMINA UMANA 200 g/l* 50 ML-1**	human albumin	supportive	0	0	0	11	11	9
**IRINOTECAN ACCORD 100 mg/5ml-2**	irinotecan	antineoplastic_cytotoxic	1	1	0	11	11	6
**OXALIPLATIN ACCORD 50 mg/10ml-1**	oxaliplatin	antineoplastic_cytotoxic	1	1	0	11	9	8
**TRAMADOL 50MG/1ML-1**	tramadol	supportive	0	0	0	11	6	3
**5 FLUOROURACIL EBEWE 1000MG 50MG/ML-3**	fluorouracil	antineoplastic_cytotoxic	1	1	0	10	10	5
**VITAMINA B1 100MG/2ML-2**	thiamine (vitamin B1)	supportive	0	0	0	10	9	6
**VITAMINA B6 250MG/ML-2**	pyridoxine (vitamin B6)	supportive	0	0	0	10	9	6
**VITAMINA C 500 mg/5ml-1**	ascorbic acid (vitamin C)	supportive	0	0	0	10	10	9
**VITAMINA C 750MG/5ML-2**	ascorbic acid (vitamin C)	supportive	0	0	0	10	7	6
**CLORURA DE SODIU 0,9%-250 ml Braun-3**	sodium chloride solution	fluid_electrolyte	0	0	0	9	5	4
**CLORURA DE SODIU 9 mg/500ML BRAUN-4**	sodium chloride solution	fluid_electrolyte	0	0	0	9	9	4
**ERBITUX 100mg/20ml-5**	cetuximab	antineoplastic_targeted	1	1	0	9	9	2
**ETOPOSIDE-TEVA 100mg/5ml-2**	etoposide	antineoplastic_cytotoxic	1	0	0	9	3	1
**FOLINAT DE CALCIU KALCEKS 100 MG/10 ML-7**	calcium folinate (leucovorin)	treatment_modulator	0	1	0	8	6	3
**FUROSEMID 20 mg/2 ml-1**	furosemide	supportive	0	0	0	8	8	7
**NO-SPA 40 mg/2 ml-2**	drotaverine	supportive	0	0	0	8	3	3
**OMEPRAZOL ROMPHARM 40 MG-3**	omeprazole	supportive	0	0	0	8	7	5
**OYAVAS 100 mg/4ml-3**	bevacizumab	antineoplastic_targeted	1	1	0	8	8	4
**VEGZELMA 100mg/4ml-6**	bevacizumab	antineoplastic_targeted	1	1	0	8	8	6
**VITAMINA C 500 mg/5ml-5**	ascorbic acid (vitamin C)	supportive	0	0	0	8	7	4
**5 FLUOROURACIL EBEWE 1000MG 50MG/ML-5**	fluorouracil	antineoplastic_cytotoxic	1	1	0	7	5	3
**ALGOCALMIN 1G/2ML*5F-2**	metamizole	supportive	0	0	0	7	4	4
**CLORURA DE SODIU 9 mg/500ML BRAUN-3**	sodium chloride solution	fluid_electrolyte	0	0	0	7	5	4
**GLUCOZA 5% 250mlx30pg+2tub-2**	glucose solution	fluid_electrolyte	0	0	0	7	7	5
**HEPAXANE 6000 UI/0.6 ML-1**	Hepaxane (LMWH trade name)	supportive	0	0	1	7	1	1

**IRINOTECAN ACCORD 100 mg/5ml-1**	irinotecan	antineoplastic_cytotoxic	1	1	0	7	5	4
**SOLUTIE RINGER 500 ML BRAUN-3**	Ringer solution	fluid_electrolyte	0	0	0	7	6	5
**VITAMINA C 500 mg/5ml-4**	ascorbic acid (vitamin C)	supportive	0	0	0	7	7	7
**CLORURA DE SODIU 0,9%-250 ml Braun-2**	sodium chloride solution	fluid_electrolyte	0	0	0	6	6	4
**FLUIMUCIL 300MG/3ML-5**	acetylcysteine	supportive	0	0	0	6	4	2
**LIDOCAINA KABI 10 mg/ml* 10 ml-1**	lidocaine	supportive	0	0	0	6	6	5
**ALGOCALMIN 1G/2ML*5F-3**	metamizole	supportive	0	0	0	5	4	3
**ASPATOFORT 10 ML-4**	Aspatofort (trade name)	supportive	0	0	1	5	5	4
**CLORURA DE SODIU 0,9% 100ML BRAUN-3**	sodium chloride solution	fluid_electrolyte	0	0	0	5	5	5
**DEXAMETHASONE SODIUM PHOSPHATE 8 mg/2 ml-3**	dexamethasone	supportive	0	0	0	5	5	4
**FLUIMUCIL 300MG/3ML-3**	acetylcysteine	supportive	0	0	0	5	5	2
**FOLINAT DE CALCIU KALCEKS 100 MG/10 ML-6**	calcium folinate (leucovorin)	treatment_modulator	0	1	0	5	5	2
**KABIVEN PERIPHEAL ,emulsie perf. 1440mlx 4 saci-1**	Kabiven parenteral nutrition	nutrition	0	0	1	5	3	2
**MANITOL INFOMED 250 ml-1**	mannitol	supportive	0	0	0	5	4	4
**METAMIZOL KALCEKS 500MG/ML *2ML-3**	metamizole	supportive	0	0	0	5	3	3
**NO-SPA 40 mg/2 ml-3**	drotaverine	supportive	0	0	0	5	4	3
**OMEPRAZOL ROMPHARM 40 MG-2**	omeprazole	supportive	0	0	0	5	5	5
**OXALIPLATIN ACCORD 100MG/20ML-2**	oxaliplatin	antineoplastic_cytotoxic	1	1	0	5	5	4
**SOLUTIE RINGER 500 ML BRAUN-2**	Ringer solution	fluid_electrolyte	0	0	0	5	5	3
**VEGZELMA 100mg/4ml-3**	bevacizumab	antineoplastic_targeted	1	1	0	5	5	4
**CLORURA DE SODIU 0,9% 100ML BRAUN-2**	sodium chloride solution	fluid_electrolyte	0	0	0	4	3	3
**IRINOTECAN KABI 100 MG 20 mg/ml-1**	irinotecan	antineoplastic_cytotoxic	1	1	0	4	4	3
**IRINOTECAN KABI 100 MG 20 mg/ml-2**	irinotecan	antineoplastic_cytotoxic	1	1	0	4	4	4
**MANITOL 500 ML 200MG/ML-1**	mannitol	supportive	0	0	0	4	4	4
**OXALIPLATIN KABI 100MG 5 mg/ml-2**	oxaliplatin	antineoplastic_cytotoxic	1	1	0	4	4	2
**OYAVAS 100 mg/4ml-2**	bevacizumab	antineoplastic_targeted	1	1	0	4	4	2
**TRAMADOL 50MG/1ML-2**	tramadol	supportive	0	0	0	4	3	3
**VITAMINA B1 100MG/2ML-3**	thiamine (vitamin B1)	supportive	0	0	0	4	4	3
**VITAMINA B6 250MG/ML-3**	pyridoxine (vitamin B6)	supportive	0	0	0	4	4	3
**AMINOSTERIL N-HEPA 8% SOL.PERF.500ML-1**	Aminosteril amino-acid solution	nutrition	0	0	1	3	2	2
**ASPATOFORT 10 ML-1**	Aspatofort (trade name)	supportive	0	0	1	3	3	2
**CLORURA DE SODIU 0,9% 100ML BRAUN-4**	sodium chloride solution	fluid_electrolyte	0	0	0	3	3	3

**CLORURA DE SODIU 0.9% 250 ml-4**	sodium chloride solution	fluid_electrolyte	0	0	0	3	3	3
**ERBITUX 100mg/20ml-7**	cetuximab	antineoplastic_targeted	1	1	0	3	3	1
**FLUIMUCIL 300MG/3ML-1**	acetylcysteine	supportive	0	0	0	3	3	1
**METOCLOPRAMID TERAPIA 5mg/ml* 2 ML-3**	metoclopramide	supportive	0	0	0	3	1	1
**OYAVAS 100 mg/4ml-1**	bevacizumab	antineoplastic_targeted	1	1	0	3	3	2
**OYAVAS 100 mg/4ml-6**	bevacizumab	antineoplastic_targeted	1	1	0	3	3	2
**OYAVAS 400 mg/16 ml-2**	bevacizumab	antineoplastic_targeted	1	1	0	3	3	1
**SMOFlipid 200g/1000ml-1**	SMOFlipid lipid emulsion	nutrition	0	0	1	3	2	2
**VECTIBIX 100mg/5ml-3**	panitumumab	antineoplastic_targeted	1	1	0	3	3	1
**VEGZELMA 100mg/4ml-7**	bevacizumab	antineoplastic_targeted	1	1	0	3	3	2
**ALBUMINA UMANA 200 g/l* 50 ML-2**	human albumin	supportive	0	0	0	2	2	2
**ALGOCALMIN 1G/2ML*5F-4**	metamizole	supportive	0	0	0	2	2	2
**ARGININA SORBITOL* 500 ml-1**	arginine/sorbitol solution	supportive	0	0	0	2	1	1
**CARBOPLATIN KABI 450 mg/45ml-1**	carboplatin	antineoplastic_cytotoxic	1	0	0	2	2	1
**CEFORT 1G *10 fl*15 ml-2**	Cefort (antibacterial trade name)	supportive	0	0	1	2	1	1
**DEXAMETHASONE SODIUM PHOSPHATE 8 mg/2 ml-4**	dexamethasone	supportive	0	0	0	2	2	2
**DEXAMETHASONE SODIUM PHOSPHATE 8 mg/2 ml-6**	dexamethasone	supportive	0	0	0	2	2	2
**FAMOTIDINA ZENTIVA 40 mg-1**	famotidine	supportive	0	0	0	2	2	2
**FUROSEMID 20 mg/2 ml-2**	furosemide	supportive	0	0	0	2	2	1
**GLUCOZA 5% 500 ML STADA-3**	glucose solution	fluid_electrolyte	0	0	0	2	2	1
**HIDROCORTIZON HF 100 mg-HF-2**	hydrocortisone	supportive	0	0	0	2	1	1
**LEMOD SOLU 125 MG-1**	Lemod Solu (corticosteroid trade name)	supportive	0	0	1	2	2	2
**PANTOPRAZOL ROMPHARM 40 MG-3**	pantoprazole	supportive	0	0	0	2	2	2
**VECTIBIX 100mg/5ml-1**	panitumumab	antineoplastic_targeted	1	1	0	2	2	1
**VECTIBIX 400mg/20ml-1**	panitumumab	antineoplastic_targeted	1	1	0	2	2	1
**VENOFER 100mg fer/5ml-1**	iron sucrose	supportive	0	0	0	2	2	2
**VITAMINA C 500 mg/5ml-6**	ascorbic acid (vitamin C)	supportive	0	0	0	2	2	2
**VITAMINA C 750MG/5ML-6**	ascorbic acid (vitamin C)	supportive	0	0	0	2	2	2
**ADRENOSTAZIN 1,5mg/5ml-1**	Adrenostazin (trade name)	supportive	0	0	1	1	1	1
**ADRENOSTAZIN 1,5mg/5ml-2**	Adrenostazin (trade name)	supportive	0	0	1	1	1	1
**ARGININA SORBITOL*250ML-2**	arginine/sorbitol solution	supportive	0	0	0	1	1	1
**ARGININA SORBITOL 500ML-1**	arginine/sorbitol solution	supportive	0	0	0	1	1	1
**BETALOC 5mg/5ml-1**	metoprolol	supportive	0	0	0	1	1	1
**CARBOPLATIN TEVA 450mg 10mg/ml-1**	carboplatin	antineoplastic_cytotoxic	1	0	0	1	1	1

**CEFORT 1G *10 fl*15 ml-4**	Cefort (antibacterial trade name)	supportive	0	0	1	1	1	1
**CEFORT 2 g * 10 fl-1**	Cefort (antibacterial trade name)	supportive	0	0	1	1	1	1
**CEFORT 2 g * 10 fl-2**	Cefort (antibacterial trade name)	supportive	0	0	1	1	1	1
**CLORURA DE SODIU 0,9% 100ML BRAUN-9**	sodium chloride solution	fluid_electrolyte	0	0	0	1	1	1
**CLORURA DE SODIU 0,9%-250 ml Braun-4**	sodium chloride solution	fluid_electrolyte	0	0	0	1	1	1
**DEXKETOPROFEN ROMPHARM 50 mg/2 ml-3**	dexketoprofen	supportive	0	0	0	1	1	1
**ENAP 1.25MG/ML-1**	enalapril	supportive	0	0	0	1	1	1
**ERBITUX 100mg/20ml-4**	cetuximab	antineoplastic_targeted	1	1	0	1	1	1
**ETAMSILAT 250MG-1**	etamsylate	supportive	0	0	0	1	1	1
**ETAMSILAT 250MG-2**	etamsylate	supportive	0	0	0	1	1	1
**FAMOTIDINA ZENTIVA 20mg-1**	famotidine	supportive	0	0	0	1	1	1
**FITOMENADION 10MG/ML-1**	phytomenadione	supportive	0	0	0	1	1	1
**FITOMENADION 10MG/ML-2**	phytomenadione	supportive	0	0	0	1	1	1
**FLUIMUCIL 300MG/3ML-4**	acetylcysteine	supportive	0	0	0	1	1	1
**FLUIMUCIL 300MG/3ML-6**	acetylcysteine	supportive	0	0	0	1	1	1
**FOLINAT DE CALCIU KALCEKS 100 MG/10 ML-1**	calcium folinate (leucovorin)	treatment_modulator	0	1	0	1	1	1
**FOLINAT DE CALCIU KALCEKS 100 MG/10 ML-5**	calcium folinate (leucovorin)	treatment_modulator	0	1	0	1	1	1
**GEMCITABINA KABI 1000 mg* 38 mg/ml-1**	gemcitabine	antineoplastic_cytotoxic	1	0	0	1	1	1
**GLUCONAT DE CALCIU 95MG/ML-1**	calcium gluconate	fluid_electrolyte	0	0	0	1	1	1
**GLUCOZA 5% 500 ML STADA-2**	glucose solution	fluid_electrolyte	0	0	0	1	1	1
**GRANISETRON KABI 1mg/ml 3MG/3ML-2**	granisetron	supportive	0	0	0	1	1	1
**GRANISETRON KABI 1mg/ml 3MG/3ML-3**	granisetron	supportive	0	0	0	1	1	1
**HEPAXANE 6000 UI/0.6 ML-2**	Hepaxane (LMWH trade name)	supportive	0	0	1	1	1	1
**HEPAXANE 6000 UI/0.6 ML-3**	Hepaxane (LMWH trade name)	supportive	0	0	1	1	1	1
**IRINOTECAN KABI 100 MG 20 mg/ml-3**	irinotecan	antineoplastic_cytotoxic	1	1	0	1	1	1
**KEYTRUDA 100 MG-2**	pembrolizumab	antineoplastic_immunotherapy	1	1	0	1	1	1
**LIDOCAINA KABI 10 mg/ml* 10 ml-2**	lidocaine	supportive	0	0	0	1	1	1
**NO-SPA 40 mg/2 ml-4**	drotaverine	supportive	0	0	0	1	1	1
**OMEPRAZOL ROMPHARM 40 MG-4**	omeprazole	supportive	0	0	0	1	1	1
**OYAVAS 100 mg/4ml-5**	bevacizumab	antineoplastic_targeted	1	1	0	1	1	1
**PANTOPRAZOL ROMPHARM 40 MG-4**	pantoprazole	supportive	0	0	0	1	1	1
**PARACETAMOL KABI 10 mg/ml*100 ML-3**	paracetamol	supportive	0	0	0	1	1	1
**SOLUTIE RINGER 500 ML BRAUN-4**	Ringer solution	fluid_electrolyte	0	0	0	1	1	1
**TRAMADOL 50MG/1ML-3**	tramadol	supportive	0	0	0	1	1	1
**TRAMADOL 50MG/1ML-5**	tramadol	supportive	0	0	0	1	1	1
**VENOFER 100mg fer/5ml-4**	iron sucrose	supportive	0	0	0	1	1	1

**VENOFER 100mg fer/5ml-6**	iron sucrose	supportive	0	0	0	1	1	1
**VITAMINA B1 100MG/2ML-4**	thiamine (vitamin B1)	supportive	0	0	0	1	1	1
**VITAMINA B6 250MG/ML-4**	pyridoxine (vitamin B6)	supportive	0	0	0	1	1	1
**VITAMINA C 750MG/5ML-1**	ascorbic acid (vitamin C)	supportive	0	0	0	1	1	1
**VITAMINA C 750MG/5ML-4**	ascorbic acid (vitamin C)	supportive	0	0	0	1	1	1
**VITAMINA C 750MG/5ML-5**	ascorbic acid (vitamin C)	supportive	0	0	0	1	1	1

The complete 186-entry raw-token dictionary is provided in Table S2. Rule-based mappings were reviewed by an oncologist. Ambiguous supportive mappings were not classified as antineoplastic; review of the dictionary does not independently establish that every component was administered or completely captured.

Table S3. Complete agreement metrics with patient-cluster bootstrap confidence intervals.

Analysis	Level	N	Both +	Procedure + / Medication -	Procedure - / Medication +	Both -	Agreement %	Agreement 95% CI	Kappa	Kappa 95% CI	Positive agreement %	Positive 95% CI	Negative agreement %	Negative 95% CI	Exact McNemar p
**Primary complete-case**	Encounter	891	124	0	1	766	99.888	99.64-100.00	0.9953	0.9839-1.0000	99.598	98.59-100.00	99.935	99.79-100.00	1.0
**CRC-relevant agents**	Encounter	891	120	4	1	766	99.439	98.58-100.00	0.9763	0.9385-1.0000	97.959	94.69-100.00	99.675	99.18-100.00	0.375
**Broad procedure**	Encounter	891	124	5	1	761	99.327	98.65-99.88	0.9725	0.9430-0.9950	97.638	95.04-99.57	99.607	99.21-99.93	0.21875
**Full-cohort operational**	Encounter	1310	124	0	1	1185	99.924	99.76-100.00	0.9956	0.9846-1.0000	99.598	98.59-100.00	99.958	99.87-100.00	1.0
**Patient: R001 vs any antineoplastic**	Patient	400	53	0	1	346	99.75	99.25-100.00	0.9892	0.9635-1.0000	99.065	96.77-100.00	99.856	99.55-100.00	1.0
**Patient: R001 vs CRC-relevant**	Patient	400	51	2	1	346	99.25	98.25-100.00	0.9671	0.9220-1.0000	97.143	93.20-100.00	99.568	99.00-100.00	1.0
**Patient: broad procedure vs any antineoplastic**	Patient	400	53	4	1	342	98.75	97.50-99.75	0.9477	0.8962-0.9892	95.495	90.91-99.07	99.274	98.55-99.86	0.375

Table S4. Missing-data and completeness audit.

Variable/indicator	Present/positive n	Missing/negative n	Interpretation
**Medication field**	891	419	68.0% available; blank not equivalent to no treatment
**Age at first encounter**	625	2	Two ages unresolvable; not imputed

**procprinc**	0	1310	Completely blank; procedure source was proc Sec
**proc Sec**	1310	0	Procedure source used for analysis
**Prescription raw field**	36	1274	Only derived oral-antineoplastic flags used in analysis
**R001 among medication-blank encounters**	0	419	0/419; critical selection-bias audit
**R003 among medication-blank encounters**	3	416	Planning-family codes only
**SPT_3.7 among medication-blank encounters**	0	419	None observed

Table S5. Sensitivity analyses.

Analysis	N	2 x 2 counts	Result	Interpretation
**CRC-relevant agent definition**	891	120/4/1/766	kappa 0.976 (0.938-1.000)	Unexpected antineoplastic agents excluded from CRC-relevant set.
**Broad procedure definition**	891	124/5/1/761	kappa 0.972 (0.943-0.995)	Planning/service-context codes create additional procedure-only classifications.
**Operational blank-as-negative**	1310	124/0/1/1185	kappa 0.996 (0.985-1.000)	Scenario only; does not assert treatment absence.
**Patient-level primary**	400	53/0/1/346	kappa 0.989 (0.964-1.000)	Patients with at least one medication-available encounter.

Table S6. Descriptive medication components in adjacent encounters.

Measure	Encounters, n	Patients, n	Interval, days
**Single-agent medication-positive encounters**	64	32	Not applicable
**Different CRC component in the preceding encounter**	2	2	14 or 20
**Different CRC component in the following encounter**	1	1	14
**Different CRC component in either neighbour**	2	2	14 or 20
**Fluorouracil in a neighbour, absent at the index encounter**	1	1	14
**Directional links with a different CRC component**	3 links	2	14, 14, 20

The index set comprises 64 encounters with exactly one named antineoplastic in the medication field, among 125 medication-positive encounters. Neighbours are the immediately preceding and following observed encounters for the same patient, considering all encounters in the study window. A different component is a CRC-relevant medication class absent at the index encounter and present in a neighbour; oral prescriptions are excluded. The preceding and following counts overlap, so they must not be summed as unique index encounters. No maximum interval was imposed. The three directional links involve two index encounters and two patients; they do not establish pharmacological complementarity, components of the same cycle, or omitted administration. The result was unchanged when neighbours were restricted to medication-positive encounters. These additional calculations used Python 3.12.14 and pandas 2.2.3.

Table S7. Temporal stability.

Metric	March 2026	April 2026
**All encounters**	509	801
**Medication available**	324	567
**Medication availability**	63.7%	70.8%
**R001 positive**	67	57
**Named antineoplastic positive**	68	57
**Conditional agreement**	99.69%	100.00%
**Conditional kappa**	0.991	1.000

Table S8. Data-consistency and source-complementarity findings.

Finding	Value	Details
**C18/C19/C20 inconsistency**	18	14 C18/C20; 2 C18/C19; 2 C19/C20
**Patient site tie requiring earliest-encounter tie-break**	8	Equal modal counts
**Oral antineoplastic prescription-positive encounters**	7	4 capecitabine; 3 trifluridine-tipiracil; 0 regorafenib
**Prescription-positive episodes adding new binary treatment-positive encounter**	0	All seven were already medication-antineoplastic positive
**Primary discordant episode**	1	Bevacizumab in medication; trifluridine-tipiracil in prescription; no R001
**Unexpected antineoplastic encounters**	4	Carboplatin+etoposide: 3 encounters/1 patient; gemcitabine: 1 encounter/1 patient
**Leave-one-patient-out primary kappa range**	0.9952-1.000	Removing the sole discordant patient produces perfect agreement

Table S9. Within-patient medication-field availability patterns and consecutive-encounter transitions.

Analysis	Category	n / denominator	%
**Repeat-patient pattern**	Always available	148 / 278	53.2
**Repeat-patient pattern**	Mixed availability	103 / 278	37.1
**Repeat-patient pattern**	Never available	27 / 278	9.7
**Consecutive transition**	Available after previously available	465 / 524	88.7
**Consecutive transition**	Available after previously unavailable	83 / 159	52.2
**Patient-level opportunity**	Any medication documentation with 1 total encounter	149 / 349	42.7

**Patient-level opportunity**	Any medication documentation with 2 total encounters	128 / 153	83.7
**Patient-level opportunity**	Any medication documentation with 3 total encounters	46 / 47	97.9
**Patient-level opportunity**	Any medication documentation with >=4 total encounters	77 / 78	98.7

Table S10. Multidimensional data-quality scorecard for the systemic-therapy ascertainment use case.

Domain	Operational check	Finding	Interpretation for use case
**Conformance**	Field structure and tokenisation	procprinc blank in 1,310/1,310; proc Sec contained procedure evidence; 5,075 medication tokens from 186 distinct raw strings	Source-specific field semantics must be documented; local code details retained in Supplement
**Completeness**	Availability of key fields	Medication nonblank 891/1,310 (68.0%); age available 625/627 (99.7%); prescription field present 36/1,310 (2.7%)	Medication completeness is the principal limitation
**Concordance**	R001 vs named antineoplastic among interpretable encounters	Agreement 99.89%; kappa 0.995	Very high internal agreement, not clinical accuracy
**Plausibility**	Age/date/site checks	No discharge before admission; one age 17 retained; 18/627 patients changed C18/C19/C20 group; 15 day-hospital records had discharge after admission, 5 >1 day	Administrative semantics require caution; suspicious dates not used for LOS inference
**Temporality**	March vs April	Medication completeness 63.7% vs 70.8%; conditional kappa 0.991 vs 1.000	Short-term stability only; two months cannot establish long-term consistency

**Source overlap**	Procedure/medication/prescription	124/125 encounter positives shared by R001 and medication (Jaccard 99.2%); oral prescription added no unique binary positive encounter	Near-complete overlap suggests redundancy/shared workflow more than independent corroboration
**Robustness**	Missingness scenarios	Operational full-cohort kappa 0.996; tipping point below 0.95 at 11 hidden positives among 419 blank records	Joint undercapture remains the key unverified uncertainty

Table S11. One-sided tipping-point sensitivity analysis for hidden treatment among medication-unavailable records.

Assumed hidden antineoplastic-positive among 419 blank records	% of 419	Agreement, %	Kappa	Positive agreement, %	Negative agreement, %
**0**	0.0	99.92	0.996	99.60	99.96
**11**	2.6	99.08	0.949	95.38	99.49
**24**	5.7	98.09	0.898	90.84	98.93
**53**	12.6	95.88	0.799	82.12	97.67

This hypothetical scenario augments the medication indicator with a specified number of unobserved treatment-positive records among the 419 medication-unavailable, R001-negative encounters and compares it with R001. It leaves observed concordance unchanged and does not estimate the true missing-treatment frequency. It is an assumption-based sensitivity calculation, not an imputation model or independent clinical validation.

Cohort Reconstruction and Transformation Log

step	operation	implementation	result_or_QA
**1**	Manual source abstraction	Patient-record information entered into Excel for screening and analysis.	4,022 hospital records screened across departments and presentation types.
**2**	Eligibility filtering	Oncology, internal medicine or surgery; C18-C20 admission diagnosis; admission date >= 2026-03-01 and < 2026-05-01.	1,310 eligible encounters: 509 March, 801 April; 2,712 excluded by the combined criteria.
**3**	Primary patient linkage	National identifier (CNP) used as the primary linkage key before removal of direct identifiers.	627 unique patients.
**4**	Repeated-encounter linkage	Encounters assigned to the same patient using the national identifier, rather than names as the primary key.	1,310 encounters linked to 627 patients.
**5**	Removal of direct identifiers	Sequential study IDs and encounter IDs assigned after linkage; direct identifiers omitted from exports.	627 study IDs; 1310 encounter IDs.

**6**	Age/sex derivation	Birth date and sex derived from national-ID structure internally; century not inferred for S=7 identifiers.	Age available for 625; 2 unresolved; recorded age 17 verified by the study team.
**7**	Patient site derivation	C18/C19/C20 grouped from diagint; patient site = modal encounter group; ties resolved by earliest encounter.	C18=353; C19=32; C20=242; 18 patients had cross-encounter site inconsistency.
**8**	Medication-field status	Whitespace-trimmed blank treated as unavailable, not as no treatment.	891 nonblank; 419 blank/unavailable.
**9**	Medication tokenization	Split medication text on ';'; trim whitespace; empty tokens dropped; standalone '-N' quantity fragments merged to preceding token.	5075 tokens; 186 distinct raw tokens; two quantity fragments repaired.
**10**	Medication normalization/classification	Rule-based raw-token mapping to normalised agent/category, reviewed by an oncologist; source administration was not independently validated.	125 encounters with a named antineoplastic; 54 patients.
**11**	CRC-relevant classification	CRC-relevant antineoplastic set: fluorouracil, oxaliplatin, irinotecan, bevacizumab, cetuximab, panitumumab, pembrolizumab. Calcium folinate tracked separately as treatment modulator, not antineoplastic.	121 encounters; 52 patients.
**12**	Unexpected agents	Carboplatin, etoposide and gemcitabine retained as antineoplastic but not CRC-relevant; no indication inferred.	4 encounters across 2 patients.
**13**	Procedure parsing	procprinc + proc Sec split on comma/semicolon/newline, trimmed and deduplicated within encounter.	37 distinct procedure tokens; procprinc blank in all included encounters.
**14**	Primary procedure indicator	R001_any = any of R00101/R00102/R00103/R00107/R00108.	124 encounters; 0 among 419 medication-blank encounters.
**15**	Broad procedure indicator	R001_any OR R00301/R00302 OR SPT_3.7.	132 encounters; 3 among medication-blank encounters.
**16**	Prescription oral-agent flags	Raw prescription text screened for CAPECITABINUM, TRIFLURIDINUM+TIPIRACILUM and REGORAFENIB; raw prescription identifiers not exported.	7 encounters: 4 capecitabine, 3 trifluridine–tipiracil, 0 regorafenib.
**17**	Agreement analysis	Primary: R001 vs any named antineoplastic among medication-nonblank encounters; patient-cluster bootstrap, 10,000 resamples, seed 20260902.	2×2 = 124/0/1/766; kappa 0.99533.
**18**	Operational missingness sensitivity	All 1310 encounters included with blank medication coded negative only as a scenario analysis.	2×2 = 124/0/1/1185; kappa 0.99556. This is not a clinical no-treatment assumption.

Reproducibility Notes

The main manuscript uses medication-documented patterns as its descriptive focus and treats prescription evidence separately. Supporting analyses address missingness, source overlap, data quality, adjacent-encounter components, and hypothetical hidden treatment; they do not redefine the primary medication-only patterns. Aggregate results, the medication dictionary, and analytical definitions are included. Patient-level data, original analysis-code files and separate reporting checklists are not included; no public repository or unverified access arrangement is claimed.

Table S12. Derived-variable definitions and interpretation limits.

Variable	Level	Operational definition	Permitted interpretation	Not permitted
**First treatment-documented encounter pattern**	Patient	Medication-derived pattern at the earliest encounter within 1 March-30 April 2026 containing a named	First observed treatment-documented pattern within the fixed window.	First-line therapy or treatment initiation.

		antineoplastic medication.		
**Named antineoplastic agent count**	Encounter	Number of distinct named antineoplastic agents in the medication field; calcium folinate/leucovorin excluded.	Same-encounter component count.	Regimen name, dose intensity, or line of therapy.
**Multi-component medication documentation**	Encounter	>=2 named antineoplastic agents in the same medication field.	Multi-component same-encounter documentation.	Automatic inference of FOLFOX, FOLFIRI, CAPOX, etc.
**Medication-derived CRC-relevant class diversity**	Patient	Number of distinct medication-derived CRC-relevant classes documented in-window: fluorouracil, oxaliplatin, irinotecan, anti-VEGF, anti-EGFR, ICI.	Documented class diversity during the fixed window.	Number of treatment lines.
**Repeated treatment documentation**	Patient	>=2 encounters with named antineoplastic medication documentation.	Repeated documentation of treatment components.	Treatment cycles or treatment duration.
**Medication-derived signature stability**	Patient	Exact medication-derived class set identical across all medication-positive encounters for patients with >=2 such encounters.	Stability of the documented class signature.	Proof of regimen persistence or absence of treatment switching.
**Oral prescription evidence**	Encounter/patient	Capecitabine, trifluridine-tipiracil, or regorafenib identified in the separate prescription field.	Complementary evidence of oral treatment prescription.	Proof of administration.

**Source-support count**	Encounter	Number of positive routine data domains among R001 procedure, named antineoplastic medication, and oral antineoplastic prescription.	Multi-domain documentation support.	Independent validation or clinical accuracy.
**Unexpected-only antineoplastic flag**	Patient	Named antineoplastic medication documented but no medication-derived CRC-relevant class observed in-window.	Sensitivity flag for unusual treatment documentation.	Inference of another diagnosis or indication.

These definitions describe the reported retrospective analysis and limit inference about treatment line, regimen, duration, indication, or effectiveness; no prospective pre-specification is claimed.
